# Antibiotics, Acid and Heat Tolerance of Honey adapted *Escherichia coli*, *Salmonella* Typhi and *Klebsiella pneumoniae*

**DOI:** 10.3390/foods9030311

**Published:** 2020-03-09

**Authors:** Rabia Ayub, Muhammad Umer, Abid Aslam Maan, Bilal Rasool, Muhammad Kashif Iqbal Khan, Tahira Younis, Shabbar Abbas, Muhammad Sajjad, Imdad Kaleem, Muhammad Imran, Azmat Ullah, Muhammad Sohail Afzal, Zaheer Hussain Shah, Sheraz Ahmed, Farhan Aslam, Neelam Chaudhary, Muhammad Inam Afzal

**Affiliations:** 1 Department of Biosciences, COMSATS University Islamabad, Park road, Tarlai kalan, Islamabad 45550, Pakistan; rabiaayub54@gmail.com (R.A.); umer.imperial@gmail.com (M.U.); shabbar.abbas@comsats.edu.pk (S.A.); msajjadpbg@gmail.com (M.S.);; 2 Department of Food Engineering, University of Agriculture, Faisalabad 38000, Pakistan; abid.maan@uaf.edu.pk; 3 Department of Zoology, Faculty of Life Sciences, Government College University, Faisalabad 38000, Pakistan; bilalrasool@gcuf.edu.pk (B.R.); tahirayounis@gmail.com (T.Y.); 4 University Institute of Diet and Nutritional Sciences, Faculty of Allied Health Sciences, The University of Lahore, Lahore 54000, Pakistan; mic_1661@yahoo.com; 5 Department of Food Science and Human Nutrition, University of Veterinary and Animal Sciences, Out Fall Road, Civil Lines, Lahore 54000, Pakistan; azmat.khan@uvas.edu.pk; 6 Department of Life Sciences, University of Management and Technology, Lahore 54000, Pakistan; sohail.ncvi@gmail.com; 7 Department of Physics, University of Management and Technology, Lahore 54000, Pakistan; zaheer.hussain@umt.edu.pk; 8 Department of Food Sciences, Faculty of Bioscience, Cholistan University of Veterinary and Animal Sciences, Bahawalpur 63100, Pakistan; drsherazahmed@gmail.com; 9 Department of Food Science and Human Nutrition, University of Veterinary and Animal Sciences, Lahore 54000, Pakistan; farhanaslam111@yahoo.com; 10 Department of Continuing Education, University of Agriculture, Faisalabad 38000, Pakistan; neelam.imperial@gmail.com

**Keywords:** honey, Escherichia coli, Salmonella Typhi, Klebsiella pneumoniae, microbial adaptation, stress resistance

## Abstract

The medicinal importance of honey has been known for many decades due to its antimicrobial properties against life-threatening bacteria. However, previous studies revealed that microorganisms are able to develop adaptations after continuous exposure to antimicrobial compounds. The present study was conducted to explore the impact of subinhibitory concentrations of branded honey (Marhaba) and unbranded honey (extracted from *Ziziphus mauritiana* plant) locally available in Pakistan on *Escherichia coli* ATCC 10536, *Salmonella* Typhi and *Klebsiella pneumoniae* by investigating the development of self- or cross-resistance to antibiotics (gentamicin, kanamycin and imipenem). Minimum inhibitory concentration (MIC) and minimum bactericidal concentration (MBC) of autoclaved honeys were determined. The bacterial cells of *E. coli* ATCC 10536, *S.* Typhi and *K. pneumoniae* were subjected to honey adaptation by exposing to ¼ *×* MIC (4 passages) and ½ *×* MIC (4 passages) of both honeys. Moreover, tolerance to low pH and high temperature was also studied in adapted and unadapted cells. The decreasing trend in growth pattern (OD_600nm_) of *E. coli* ATCC 10536, *S.* Typhi and *K. pneumoniae* was observed with increases in the concentration of honeys (6.25–50% *v/v*) respectively. Our results showed that continuous exposure of both honeys did not lead to the development of any self- or cross-resistance in tested bacteria. However, percent survival to low pH was found to be significantly higher in adapted cells as compared to unadapted cells. The results indicate that both branded honey (Marhaba) and unbranded honey (extracted from *Ziziphus mauritiana* plant) were effective in controlling the growth of tested pathogenic bacteria. However, the emergence of tolerance to adverse conditions (pH 2.5, temperature 60 °C) deserves further investigation before proposing honey as a better antibacterial agent in food fabrication/processing, where low pH and high temperatures are usually implemented.

## 1. Introduction

The development of antibiotic resistance in pathogenic microorganisms is a major threat to human health which could accelerate the mortality rate. In the present era, scientists are trying to overcome this concern through the incorporation of natural compounds in foods as a supplement or medicines [1]. Nowadays, the research has rapidly focused on honey due to its natural origin, with antimicrobial, antifungal and anti-inflammatory properties [2]. Honey is a natural food that contains numerous compounds playing an important role in the development of advanced medicines [3]. 

Honey is a saccharine exudation produced by plants and gathered by different species of honeybees comprising 80–85% carbohydrates, 15–17% water, 0.1–0.4% protein, and 0.2% ash, minute quantity of amino acids, phenolic content, enzymes and vitamins [4,5]. In the US, 300 different varieties of honey are reported on the basis of different floral origin [6]. It is a great source of flavonoids, phenolic acids and antioxidants (catalase, glucose oxidase, carotenoid derivatives, organic acids, ascorbic acid, amino acids and proteins) [6]. The worldwide annual production of honey is approximately 1.2 million tons [7]. It has been used since ancient times for its nutritional as well as curative properties [8]. The use of honey as medicine started six thousand years ago [9]. Ancient Greek athletes used water and honey mixture to overcome fatigue [2]. The ancient Egyptians, Chinese, Romanians and Assyrians used honey for wound healing and treatment of gut infections [2]. The wound healing property of honey seemed to be directly linked to its antimicrobial activity [10,11]. Various types of honey were found to possess antimicrobial activity against antibiotic resistant pathogenic microorganisms [12].

Previous studies highlighted the antimicrobial activity of different types of honey in terms of their botanical sources against pathogenic microorganisms, for instance, *Staphylococcus aureus, E. coli, S.* Typhi, *Bacillus cereus, B. subtilis, Streptococcus pyogenes* and *Shigella* spp. [4,13]. The honey extracted from *Apis indica* was found to be very effective against *E. coli, S. enterica* serovar Typhi and *P. aeruginosa* isolated from urinary tract infections, skin lesions and enteric fever [10]. A high synergistic effect of honey was observed when it was added to antibiotics against gram-negative bacteria and coagulase-positive staphylococci [14]. The strong effect of honey was observed in the detachment of biofilm by using magnesium oxide present in honey [15]. A study conducted on Manuka honey showed strong abolition of *Proteus mirabilis* and *Enterobacter cloacae* biofilms [15]. 

The aim of the present study was to explore the impact of subinhibitory concentrations of branded honey (Marhaba) and unbranded honey (extracted from *Ziziphus mauritiana* plant) locally available in Pakistan on *E. coli* ATCC 10536, *S.* Typhi and *K. pneumoniae* by investigating the development of self- or cross-resistance to antibiotics (gentamicin, kanamycin and imipenem). Moreover, tolerance to low pH and high temperature was also studied in adapted and unadapted cells.

## 2. Materials and Methods 

### 2.1. Bacterial Strains and Reagents

Two bacterial pathogens including *S.* Typhi and *K. pneumoniae*, along with one reference strain, *E. coli* ATCC 10536 were used in this study. The strains and isolates were provided by Pakistan Institute of Medical Sciences (PIMS) Islamabad, Pakistan and Microbiology and Public Health Laboratory, COMSATS University Islamabad, Pakistan. Cultures were revived and grown in tryptic soy broth supplemented with yeast extract (TSB-YE) and incubated at optimum growth temperatures at 37 °C. Three antibiotics (kanamycin, imipenem and gentamicin) were purchased from Thermo Fisher Scientific UK, Merck USA and Barrett Hodgson Pakistan. 

### 2.2. Honey Collection and Storage

Branded honey (Marhaba) was purchased from a supermarket of Islamabad, Pakistan and unbranded honey (extracted from *Ziziphus mauritiana* plant) was obtained from National Agriculture Research center (NARC), Islamabad, Pakistan. The chemical composition of branded honey (Marhaba) was previously reported [16]. The honey samples were kept in tightly sealed bottles in the dark at room temperature and autoclaved at 121 °C for 15 min before antimicrobial assays. 

### 2.3. Determination of Minimum Inhibitory Concentrations (MICs) of Honeys and Antibiotics against Bacterial Isolates 

Antimicrobial activity of branded honey (Marhaba), unbranded honey (extracted from *Ziziphus mauritiana* plant) and antibiotics (kanamycin, imipenem and gentamicin) against *E. coli* ATCC 10536, *S.* Typhi and *K. pneumoniae* was explored by using agar dilution and the spot inoculation method according to Clinical and Laboratory Standards Institute (CLSI, 2012) [17], with some modifications. Different concentrations of each honey (1.56–50% *v/v*) and antibiotics including kanamycin (0.5–8 µg/mL), imipenem (0.0075–0.24 µg/mL) and gentamicin (0.5–2 µg/mL) were selected as reported previously [10,18]. The plates were then divided into different sectors for spot inoculation of strains. Prior to each experiment, all tested microorganisms were sub-cultured in TSB-YE and incubated at 37 °C for 24 h. Bacterial cell suspensions were adjusted to 0.5 McFarland equivalents in a sodium chloride solution (0.85% *w/v*) and subsequently diluted to achieve a cell suspension with approximately 1 × 10^6^ colony forming units per mL (CFU/mL). 1–2 µL of each bacterial culture was inoculated on each spot on plates and incubated at 37 °C for 18–24 h. The TSA-YE plates without honeys or antibiotics were also inoculated as a control to check the growth of bacterial strains. Samples were subjected to bacterial numeration for the assessment of viable cells for bactericidal concentration. The minimum inhibitory concentrations (MIC) and minimum bactericidal concentration (MBC) values were noted after 24 h [10]. Three biological replicates were used.

### 2.4. Determination of the Effect of Branded and Unbranded Honey on Bacterial Growth

Bacterial cultures were prepared as described above with approximately 10^6^ CFU/mL. Growth response of *E. coli* ATCC 10536, *S.* Typhi *and K. pneumoniae* in TSB-YE was evaluated by treating bacterial cultures with different concentrations of branded and unbranded honeys (0, 6.25, 12.5, 25 and 50% *v/v*) during 48 h of incubation at 37 °C. Three biological replicates were used. 

### 2.5. Adaptation to Branded and Unbranded Honey

The adaptation assays were performed by using branded and unbranded honey against *E. coli* ATCC 10536, *S.* Typhi and *K. pneumoniae* as described previously [19] with some modifications. The bacterial cells (1 × 10^8^ CFU/mL) were exposed to ¼, ½, 1 and 2 × MIC values of branded and unbranded honey respectively. 10 µL of each cell suspension (1 × 10^8^ CFU/mL) was inoculated in 6 mL TSB-YE and incubated for 24 h at 37 °C. The bacterial growth was monitored during each passage by measuring optical density (OD_600nm_) at 0 and 24 h at 37 °C of incubation using a spectrophotometer. The 24 h incubated samples (100 µL) were centrifuged (8000× *g*, 5 min at 4 °C) and supernatants were discarded. After the first passage, 1 mL of fresh TSB-YE (previously adjusted to ¼ × MIC) was added in tubes containing cell pellets. The step was repeated 4 times (4 passages) for ¼ × MIC concentration. After the fourth passage, 1 mL of fresh TSB-YE (previously adjusted to ½ × MIC) was added in tubes containing cell pellets. The step was repeated 4 times (4 passages). After the eighth passage, 1 mL of fresh TSB-YE (previously adjusted to 1 × MIC) was added in tubes containing cell pellets. The step was repeated 4 times (4 passages). After the twelfth passage, 1 mL of fresh TSB-YE (previously adjusted to 2 × MIC) was added in tubes containing cell pellets. Honey concentrations were subsequently increased until there was no cell growth. The assays were followed by a growth control with bacterial cultures being inoculated in TSB-YE without the addition of honeys. Three biological replicates were used.

### 2.6. Determination of Adaptive and Cross-Resistance in Bacterial Isolates

The adaptive and cross-resistance was evaluated in *E. coli* ATCC 10536, *S.* Typhi and *K. pneumoniae* after the 4th passage of cells against ¼ × MIC and the 4th passage with ½ × MIC respectively. Adapted cells were evaluated for MIC and MBC values of honeys using agar dilution and spot inoculation method as described above [20]. 

### 2.7. Determination of Tolerance to Acid and Heat

Tolerance to acid (pH 2.5) and heat (60 °C) was performed as described previously [21] with some modifications. Acid tolerance assays were performed by taking 25 µL of adapted and unadapted cell suspensions (10^6^ CFU/mL) and added to tubes containing 4975 µL of TSB-YE acidified to pH 2.5 using hydrochloric acid and incubated at 37 °C. Heat tolerance assays were conducted by taking 25 µL of adapted and unadapted cell suspensions (10^6^ CFU/mL) and added to tubes containing 4975 µL of TSB-YE incubated at 60 °C in a thermostatic water bath. Bacterial viable counting was performed via the pour plate method by taking samples during 0–120 min. Three independent experiments were conducted. 

### 2.8. Statistical Analysis

Mean and standard deviations were determined for each treatment intervals. Data were analyzed using one-way ANOVA, with association between treatments. The analysis was assessed for significance at *p* < 0.05 [22].

## 3. Results and Discussion

### 3.1. Minimum Inhibitory Concentrations and Effects of Honeys on Bacterial Growth 

The MIC values of branded honey were found to be 12.5% *v/v* against *S.* Typhi and 25% *v/v* against *E. coli* ATCC 10536 and *K. pneumoniae.* The MBC values of branded honey were observed as 25% *v/v* against *S.* Typhi and 50% *v/v* against *E. coli* ATCC 10536 and *K. pneumoniae.* The MIC value of unbranded honey was found to be 25% *v/v* against *S.* Typhi, *E. coli* ATCC 10536 and *K. pneumoniae.* The MBC value of unbranded honey was noted as 50% *v/v* against *S.* Typhi, *E. coli* ATCC 10536 and *K. pneumoniae.*

**Figure 1:**
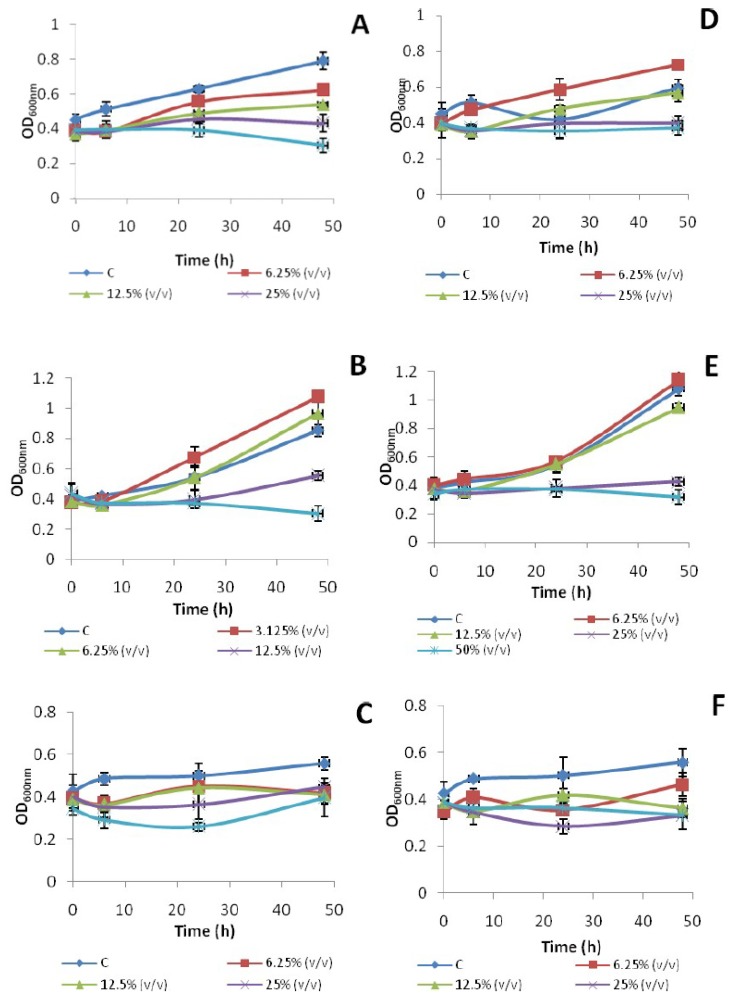
Effect of different concentrations of branded honey (Marhaba) (**A**–**C**) and unbranded honey (extracted from ***Ziziphus mauritiana*** plant) (**D**–**F**) on bacterial growth (OD_600nm_) of *E. coli* ATCC 10536 (**A**,**D**), *S.* Typhi (**B**,**E**) and *K. pneumoniae* (**C**,**F**).

Growth pattern of *E. coli* ATCC 10536, *S.* Typhi and *K. pneumoniae* treated with different concentrations of branded and unbranded honey is shown in Figure 1. All bacterial cultures grew well in the absence (control) and presence of 3.125% (*v/v*) of honeys. Branded and unbranded honey started to show growth inhibition as compared to control at 6.25% (*v/v*) for *E. coli* ATCC 10536 and *K. pneumoniae*, 12.5% (*v/v*) for *S.* Typhi. Minimum bactericidal concentrations of honeys were achieved at 25% (*v/v*) for *E. coli* ATCC 10536, *S.* Typhi and *K. pneumoniae.* A similar decreasing trend in growth was observed in *S. aureus* with increases in concentration of honeys (Manuka, Nigella and Sidr) ranging from 10 to 50% (*v/v*) in nutrient broth [18]. 

Antimicrobial activity of honeys related to coniferous, thyme and polyfloral origin against certain pathogenic microorganisms was highlighted by Olaitan and Voidarou [4,13]. The MIC of Ulmo honey was found to be 12.5% (*v/v*) for *P. aeruginosa* and *E. coli* [4,13]. The MICs of Manuka honey and Tualang honey ranged from 8.75%-25% (*v/v*) for many gram positive and gram negative pathogenic bacteria. The MICs of Nilgiri honey for *E. coli, S. aureus* and *P. aeruginosa* were noted as 40%, 35% and 25% (*v/v*), respectively [11]. 

### 3.2. Adaptation to Subinhibitory Concentrations of Branded and Unbranded Honey 

The variation in growth of *E. coli* ATCC 10536, *S.* Typhi and *K. pneumoniae* treated with ¼ *×* MIC, ½ *×* MIC, 1x MIC and 2x MIC is shown in Table 1. The exposure to subinhibitory concentrations of branded and unbranded honey induced low adaptation in *E. coli* ATCC 10536, *S.* Typhi and *K. pneumoniae*. A prominent decreased growth was observed from the fourth passage at ¼ × MIC to the first passage at ½ × MIC of branded honey in *E. coli* ATCC 10536 (2.33 ± 0.03 to 1.69 ± 0.10) and *K. pneumoniae* (2.73 ± 0.21 to 1.57 ± 0.17). However, from the second to the third passage at ½ × MIC, a significant increase in growth was observed in *K. pneumoniae* (*p* < 0.05) which might be linked to cellular changes, indicative of an adaptation to branded honey. No significant difference in growth was observed from the second to the fourth passage at ½ *×* MIC of unbranded honey in *E. coli* ATCC 10536 and *S.* Typhi. An accentuated decrease in growth was observed from the fourth passage at ¼ *×* MIC to the first passage at ½ *×* MIC of unbranded honey in *E. coli* ATCC 10536 (2.11 ± 0.06 to 1.57 ± 0.15) and *K. pneumoniae* (2.63 ± 0.06 to 1.36 ± 0.25). Nevertheless, a remarkable increase in growth was recorded in *K. pneumoniae* (*p* < 0.05) at ½ *×* MIC from the second to the third passage, which suggests that this type of cellular variation also shows adaptation to unbranded honey. The exposure to subinhibitory concentrations of honeys was found to induce low adaptation of *E. coli* ATCC 10536 and *K. pneumoniae* to the compound as observed previously by *S. aureus* against subinhibitory concentrations of resveratrol [23]. A decreasing growth trend was observed from the fourth passage at ¼ *×* MIC until the fourth passage at 1 *×* MIC for all bacteria possibly because the cells were not in a state to cope with increasing inhibitory concentrations of honeys. No growth was observed when cells were exposed to 2 *×* MIC values of honeys. The continuous exposure of bacteria to ¼ and ½ *×* MIC of honeys helped in the selection of bacterial population that became adapted or tolerant to antimicrobial compounds, possibly due to changes in over-expression and efflux pumps [24]. 

### 3.3. Impact of Honey Adaptation on the Development of Self or Cross-resistance

The MICs of branded honey remained the same in adapted and unadapted cells of *E. coli* ATCC 10536 (25% *v/v*), *S.* Typhi (12.5% *v/v*) and *K. pneumoniae* (25% *v/v*) (Table 2). Similarly, MICs of unbranded honey did not change for adapted and unadapted cells of *E. coli* ATCC 10536 (25% *v/v*), *S.* Typhi (25% *v/v*) and *K. pneumoniae* (25% *v/v*). MIC of gentamycin (2 µg/mL), kanamycin (8 µg/mL) and imipenem (0.12 µg/mL) remained the same in adapted and unadapted cells of tested bacteria. The same pattern was observed for adapted cells of *S. aureus* and *L. monocytogenes* against resveratrol, ampicillin, erythromycin, vancomycin and benzalkonium chloride [23]. On the contrary, Cooper and colleagues [25] observed a temporary increase in MIC of Manuka honey against adapted cells of *E. coli, P. aeruginosa* and *S. epidermidis* [25]. Apolónio and colleagues [19] observed no resistance development in *S. aureus* and *L. monocytogenes* when continuously exposed to subinhibitory concentrations of eugenol and citral. 

### 3.4. Influence of Honey Adaptation on the Development of Tolerance to Acid and Heat

Food products are usually treated with high temperature and low pH at the industrial scale to eliminate harmful bacteria. In order to investigate the influence of honey adaptation by bacterial cells on tolerance to acid and heat, both adapted and unadapted bacterial cells were subjected to acid (pH 2.5) and heat (60 °C) challenge assays.

**Figure 2:**
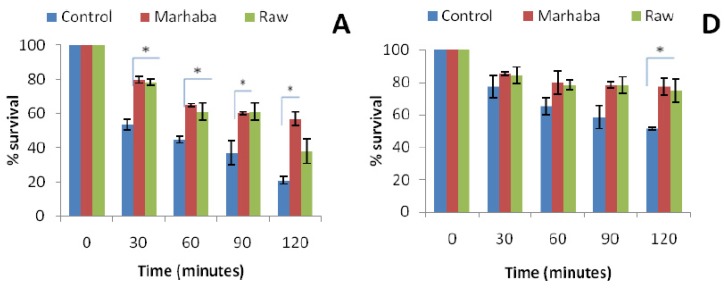

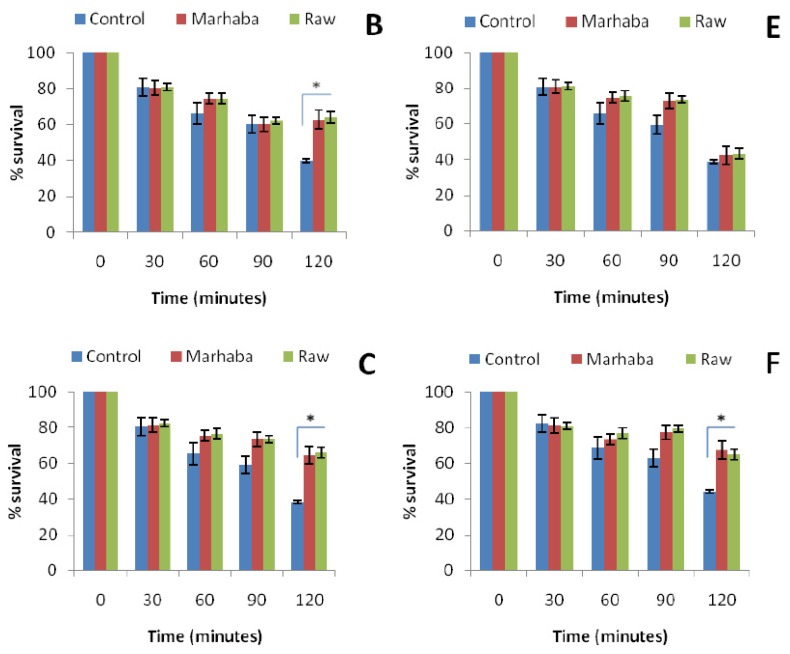
Effect of pH (2.5) in *E. coli* ATCC 10536 (**A**,**D**); *S.* Typhi (**B**,**E**) and *K. pneumoniae* (**C**,**F**) survival after the fourth passage of adaptation at ¼ *×* MIC (**A**–**C**) and the eighth until ½ *×* MIC (**D**–**F**) of branded and unbranded honey. (*p* > 0.05); * (*p* < 0.05).

The effect of the acidic environment (pH 2.5) on survival rates of *E. coli* ATCC 10536, *S.* Typhi and *K. pneumoniae* after the fourth (¼ *×* MIC) and eighth (½ *×* MIC) passage with branded and unbranded honey is shown in Figure 2. Percent survival of both adapted and unadapted cells of *E. coli* ATCC 10536 decreased during 120 min of incubation at low pH (2.5). However, percent survival of honey adapted (¼ *×* MIC) cells of *E. coli* ATCC 10536 was significantly higher (*p < 0.05) as compared to unadapted cells after 30, 60, 90 and 120 min of incubation (Figure 2A).

**Figure 3:**
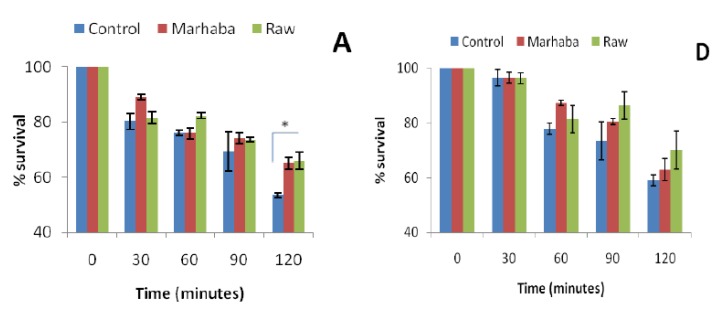

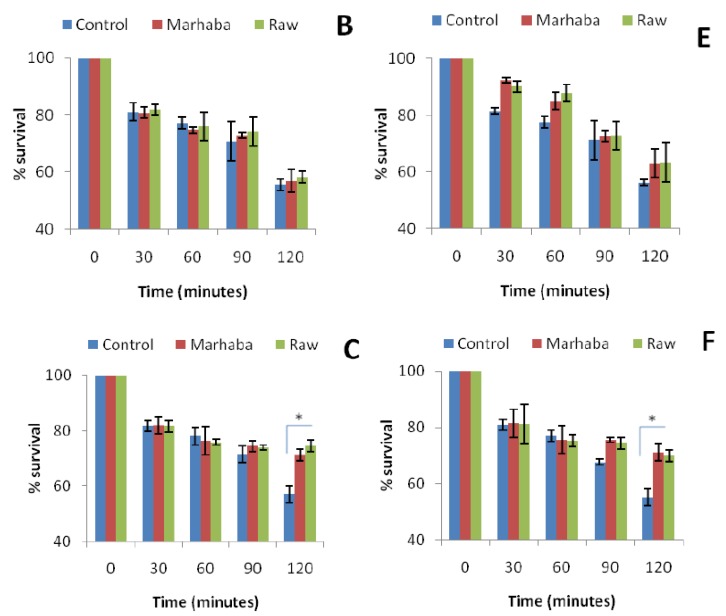
Effect of temperature (60 °C) in *E. coli* ATCC 10536 (**A**,**D**); *S.* Typhi (**B**,**E**) and *K. pneumoniae* (**C**,**F**) survival after the fourth passage of adaptation at ¼ *×* MIC (**A**–**C**) and the eighth until ½ *×* MIC (**D**–**F**) of branded and unbranded honey. (*p* > 0.05); * (*p* < 0.05).

No significant difference (*p* > 0.05) was observed for honey adapted (½ *×* MIC) *E. coli* ATCC 10536 during 0, 30, 60 and 90 min except after 120 min (Figure 3D). Percent survival of honey adapted (¼ *×* MIC) cells of *S.* Typhi and *K. pneumoniae* was found to be significantly higher (*p < 0.05) as compared to unadapted cells after 120 min of incubation (Figure 2B,C,F). No significant increase in percent survival of honey adapted (½ *×* MIC) *K. pneumoniae* was noted during 120 min of incubation. A comparable pattern was previously reported with resveratrol, carvacrol and oregano essential oil adapted cells of *S. aureus*, *L. monocytogenes* under low pH (2.4) conditions [23,26].

The effect of heat stress (60 °C) on survival rates of *E. coli* ATCC 10536, *S.* Typhi and *K. pneumoniae* after fourth (¼ *×* MIC) and eighth ½ *×* MIC) passage with branded and unbranded honey is represented in Figure 3. A significant increase in percent survival was only seen in branded and unbranded honey adapted cells of *E. coli* ATCC 10536 and *K. pneumoniae* after 120 min of incubation at 60 °C (Figure 3A,C,F).

No significant difference in the percent survival of honey adapted cells was observed as compared to unadapted cells during 0, 30, 60 and 90 min of survival assays. Furthermore, a reduction in cell viability was found during 120 min of incubation at 60 °C. This reduction in cell viability could be related to proteins involved in cellular functions being inactivated to heat shock as reported previously [27]. Heat tolerance was previously observed in resveratrol adapted *L. monocytogenes* and *S. aureus* cells during incubation at 55 °C [23].

## 4. Conclusions

The development of novel antimicrobial strategies in foods is of prior importance to the food industry to ensure safe food to consumers. However, it is also important to understand the capability of microorganisms to develop resistance against sub-lethal and lethal inhibitory concentrations of antimicrobial compounds. Our results revealed that both branded honey (Marhaba) and unbranded honey (extracted from *Ziziphus mauritiana* plant) were effective in controlling the growth of tested pathogenic bacteria. No emergence of self or cross-resistance was observed in adapted cells. However, the emergence of tolerance to high temperature (60 °C) and low pH (2.5) in adapted cells deserves further investigation, before proposing honey as a better antibacterial agent in food fabrication/processing, where low pH and high temperatures are usually implemented. 

## Figures and Tables

**Table 1:** Variation in growth of *E. coli* ATCC 10536, *S.* Typhi and *K. pneumoniae* at subinhibitory and inhibitory concentrations of branded and unbranded honey.

Preservative Concentration/Passage	*E. coli* ATCC 10536				*S.* Typhi				*K. pneumoniae*			
	¼ *×* MIC	½ *×* MIC	1 *×* MIC	2 *×* MIC	¼ *×* MIC	½ *×* MIC	1 *×* MIC	2 *×* MIC	¼ *×* MIC	½ *×* MIC	1 *×* MIC	2 *×* MIC
**Control**												
**1st**	2.23 ± 0.15 ^a^	1.81 ± 0.51 ^a^	1.21 ± 0.21 ^a^	1.92 ± 0.65	2.15 ± 0.02 ^a^	1.85 ± 0.25 ^a^	1.15 ± 0.16 ^a^	1.41 ± 0.12	2.32 ± 0.26 ^a^	1.81 ± 0.02 ^a^	1.29 ± 0.28 ^a^	2.22 ± 0.45
**2nd**	2.44 ± 0.23 ^a^	1.96 ± 0.68 ^b^	1.75 ± 0.65 ^b^		1.81 ± 0.14 ^b^	1.92 ± 0.14 ^a^	1.52 ± 0.03 ^b^		1.85 ± 0.14 ^b^	1.88 ± 0.08 ^a^	1.81 ± 0.15 ^b^	
**3rd**	2.15 ± 0.35 ^a^	1.35 ± 0.85 ^c^	1.69 ± 0.45 ^c^		2.36 ± 0.05 ^a c^	1.30 ± 0.04 ^bc^	1.53 ± 0.22 ^bc^		2.71 ± 0.08 ^c^	1.24 ± 0.15 ^b^	1.32 ± 0.06 ^ac^	
**4th**	2.44 ± 0.08 ^a^	1.52 ± 0.64 ^d^	1.86 ± 0.85 ^d^		2.52 ± 0.21 ^c^	1.41 ± 0.07 ^c^	1.48 ± 0.13 ^bcd^		2.10 ± 0.15 ^ab^	1.53 ± 0.18 ^c^	1.61 ± 0.01^bc^	
**Branded honey**												
**1st**	2.58 ± 0.05 ^a^	1.69 ± 0.10 ^a^	1.21 ± 0.04 ^a^	NG *	1.43 ± 0.31 ^a^	1.53 ± 0.10 ^a^	1.10 ± 0.21 ^a^	NG *	1.84 ± 0.04 ^a^	1.57 ± 0.17 ^a^	1.08 ± 0.12 ^a^	NG*
**2nd**	1.82 ± 0.09 ^a^	1.54 ± 0.01 ^a^	1.09 ± 0.08 ^b^		1.39 ± 0.05 ^a^	1.03 ± 0.24 ^b^	1.65 ± 0.07 ^b^		1.89 ± 0.09 ^a^	1.24 ± 0.11 ^b^	1.04 ± 0.05 ^a^	
**3rd**	1.57 ± 0.01 ^a^	1.70 ± 0.05 ^a^	1.27 ± 0.03 ^a^		1.49 ± 0.08 ^a^	1.18 ± 0.05 ^bc^	1.41 ± 0.06 ^bc^		0.86 ± 0.15 ^b^	1.74 ± 0.03 ^a^	1.24 ± 0.30 ^a^	
**4th**	2.33 ± 0.03 ^a^	1.26 ± 0.12 ^b^	1.11 ± 0.15 ^a^		1.53 ± 0.04 ^a^	1.09 ± 0.09 ^bcd^	1.02 ± 0.01 ^a^		2.73 ± 0.21 ^c^	1.25 ± 0.14 ^bc^	1.20 ± 0.21 ^a^	
**Unbranded honey**												
**1st**	2.61 ± 0.02 ^a^	1.57 ± 0.15 ^a^	1.14 ± 0.05 ^a^	NG *	1.32 ± 0.05 ^a^	1.37 ± 0.14 ^a^	1.00 ± 0.12 ^a^	NG *	2.15 ± 0.07 ^a^	1.36 ± 0.25 ^a^	0.96 ± 0.02 ^a^	NG*
**2nd**	1.65 ± 0.08 ^a^	1.22 ± 0.04 ^a^	1.25 ± 0.14 ^a^		1.63 ± 0.15 ^b^	1.29 ± 0.16 ^a^	1.12 ± 0.18 ^a^		1.94 ± 0.15 ^b^	1.33 ± 0.14 ^a^	1.08 ± 0.03 ^ac^	
**3rd**	1.31 ± 0.01 ^b^	1.55 ± 0.19 ^a^	1.12 ± 0.03 ^a^		1.80 ± 0.17 ^bc^	1.43 ± 0.05 ^a^	1.30 ± 0.09 ^a^		1.64 ± 0.02 ^c^	1.44 ± 0.06 ^a^	1.27 ± 0.10 ^b^	
**4th**	2.11 ± 0.06 ^c^	1.53 ± 0.02 ^a^	1.29 ± 0.01 ^a^		2.18 ± 0.06 ^d^	1.61 ± 0.08 ^a^	1.12 ± 0.04 ^a^		2.63 ± 0.06 ^d^	1.29 ± 0.08 ^a^	1.17 ± 0.08 ^bc^	

* Growth variation after each passage in shown as the ration between OD_600nm_ at T_24_ and OD_600nm_ at T_0_ (ODT_24_/ODT_0_). NG: no growth. Data are representative of three replicates ± SD. (Branded honey against *E. coli* and *K. pneumoniae*: ¼ *×* MIC = 6.25%*v/v*; ½ *×* MIC = 12.5%*v/v*; 1 *×* MIC = 25%*v/v*; 2 *×* MIC = 50%*v/v*). (Branded honey against *S.* Typhi: ¼ *×* MIC = 3.125%*v/v*; ½ *×* MIC = 6.25%*v/v*; 1 *×* MIC = 12.5%*v/v*; 2 *×* MIC = 25%*v/v*). (Unbranded honey against *E. coli*, *S.* Typhi and *K. pneumoniae*: ¼ *×* MIC = 6.25%*v/v*; ½ *×* MIC = 12.5%*v/v*; 1 *×* MIC = 25%*v/v*; 2 *×* MIC = 50%*v/v*). Data in columns with different superscript letters are significantly different (*p* < 0.05).

**Table 2:** Minimum inhibitory concentration (MIC) (% *v/v*) of branded and unbranded honey against *E. coli* ATCC 10536, *S.* Typhi and *K. pneumoniae* after fourth passage of the cells at ¼ and ½ *×* MIC of honeys (C: control; M: assay with branded honey; R: assay with unbranded honey.

Minimum Inhibitory Concentration (% *v/v*; µg/mL)																		
Antimicrobial Agent	*E. coli* ATCC 10536						*S.* Typhi						*K. pneumoniae*					
	4th P at ¼ *×* MIC			4th P at ½ *×* MIC			4th P at ¼ *×* MIC			4th P at ½ *×* MIC			4th P at ¼ *×* MIC			4th P at ½ *×* MIC		
	C	M	R	C	M	R	C	M	R	C	M	R	C	M	R	C	M	R
**Branded honey (% *v/v*)**	25	25	25	25	25	25	12.5	12.5	12.5	12.5	12.5	12.5	25	25	25	25	25	25
**Unbranded honey (% *v/v*)**	25	25	25	25	25	25	25	25	25	25	25	25	25	25	25	25	25	25
**Gentamicin (µg/mL)**	2	2	2	2	2	2	2	2	2	2	2	2	2	2	2	2	2	2
**Kanamycin (µg/mL)**	8	8	8	8	8	8	8	8	8	8	8	8	8	8	8	8	8	8
**Imipenem (µg/mL)**	0.12	0.12	0.12	0.12	0.12	0.12	0.12	0.12	0.12	0.12	0.12	0.12	0.12	0.12	0.12	0.12	0.12	0.12
